# Protocol to measure validity and reliability of colorectal, breast, cervical and lung cancer screening questions from the 2021 National Health Interview Survey: Methodology and design

**DOI:** 10.1371/journal.pone.0297773

**Published:** 2024-03-04

**Authors:** Larry G. Kessler, Bryan Comstock, Erin J. Aiello Bowles, Jin Mou, Michael G. Nash, Perla Bravo, Lynn E. Fleckenstein, Chaya Pflugeisen, Hongyuan Gao, Rachel L. Winer, India J. Ornelas, Cynthia Smith, Chris Neslund-Dudas, Punith Shetty

**Affiliations:** 1 Department of Health Systems and Population Health, School of Public Health, University of Washington, Seattle, Washington, United States of America; 2 Department of Biostatistics, School of Public Health, University of Washington, Seattle, Washington, United States of America; 3 Kaiser Permanente Washington Health Research Institute, Kaiser Permanente Washington, Seattle, Washington, United States of America; 4 Institute for Research and Innovation, MultiCare Health System, Tacoma, Washington, United State of America; 5 Department of Epidemiology, School of Public Health, University of Washington, Seattle, Washington, United States of America; 6 Department of Public Health Sciences, Henry Ford Hospital, Detroit, Michigan, United States of America; Centro per lo Studio e la Prevenzione Oncologica, ITALY

## Abstract

Previous studies demonstrate that self-reports of mammography screening for breast cancer and colonoscopy screening for colorectal cancer demonstrate concordance, based on adherence to screening guidelines, with electronic medical records (EMRs) in over 90% of those interviewed, as well as high sensitivity and specificity, and can be used for monitoring our Healthy People goals. However, for screening tests for cervical and lung cancers, and for various sub-populations, concordance between self-report and EMRs has been noticeably lower with poor sensitivity or specificity. This study aims to test the validity and reliability of lung, colorectal, cervical, and breast cancer screening questions from the 2021 and 2022 National Health Interview Survey (NHIS). We present the protocol for a study designed to measure the validity and reliability of the NHIS cancer screening questions compared to EMRs from four US-based healthcare systems. We planned a randomized trial of a phone- vs web-based survey with NHIS questions that were previously revised based on extensive cognitive interviewing. Our planned sample size will be 1576 validity interviews, and 1260 interviews randomly assigned at 1 or 3 months after the initial interview. We are enrolling people eligible for cancer screening based on age, sex, and smoking history per US Preventive Services Task Force recommendations. We will evaluate question validity using concordance, sensitivity, specificity, positive predictive value, negative predictive value, and report-to-records ratio. We further are randomizing participants to complete a second survey 1 vs 3 months later to assess question reliability. We suggest that typical measures of concordance may need to be reconsidered in evaluating cancer screening questions.

## Introduction

Cancer was the second leading cause of death, after heart disease, in the United States (U.S) in 2022 [1]. Although cancer related deaths decreased by 27% in the past twenty years, over 600,000 people living in the U.S. die each year of cancer [2]. An important and evidence-based intervention to help reduce cancer mortality and morbidity is timely cancer screenings for cancer types where there is evidence of benefit, usually reduced cancer-specific mortality, and consensus on screening programs. Routine cancer screenings can lead to early cancer detection for some cancers, notably cervical, breast, colorectal, and lung cancers, before symptoms appear and when cancer is most treatable [3].

For the past four decades, the U.S. Department of Health and Human Services (US DHHS) has developed measurable public health objectives every ten years, known as the Healthy People objectives. In Healthy People 2030, the objectives focus on promoting evidence-based cancer screening and prevention strategies [4]. Determining whether the US population meets these goals is a critical step in developing interventions to increase the appropriate use of cancer screenings [5]. To measure progress towards the Healthy People objectives, the US DHHS utilizes data from the National Health Interview Survey (NHIS), a nationwide in-person survey of the civilian non-institutionalized population. The sample size of the NHIS has varied considerably. For example, in 2018, the NHIS contained 25,417 Sample Adults and 8,269 Sample Children. With the 2019 redesign, an estimated 28,000 Sample Adult and 8,400 Sample Child interviews are expected to be available annually for analysis in NHIS. Since 1957, the NHIS has monitored the health of people living in the US through the collection and analysis of data on a broad range of health topics, including cancer screening utilization [6]. In addition to NHIS core questions that are asked each year, supplemental questions on cancer screening and cancer risk factors have been sponsored on the NHIS by the Division of Cancer Control and Population Sciences, National Cancer Institute (NCI) and the CDC National Center for Health Statistics (NCHS) since 1987 [7–10].

A redesigned NHIS questionnaire with new content and structure was implemented starting in January 2019 to better meet the needs of data users and to minimize respondent burden. In the redesigned NHIS (2019 or later), one sample adult and one sample child are randomly selected from each household. Prior to 2019, each family was identified within the household and then one sample adult and one child were randomly selected from each family. As part of the redesign, the cancer screening questions sponsored by the NCI and CDC were included as rotating modules on different cancer topics that periodically appear in one or more years, but not every year [11]. The total length of each annual sponsored cancer supplement is 5 minutes. The schedule for cancer topics in the rotating modules is at: https://healthcaredelivery.cancer.gov/nhis/.

To ensure that data used to measure progress towards Healthy People goals are accurate, this study plans to assess the validity and reliability of cancer screening questions on cervical, colorectal, breast and lung cancer screening from the 2021and 2022 National Health Interview Survey [12]. These screening tests are currently recommended (grade A or B) by the US Preventive Services Taskforce (USPSTF) [13]. Understanding the use of cancer screening tests among different populations is vital for planning public health interventions with the potential to increase screening uptake and reduce disparities in cancer morbidity and mortality [14]. The results of this study will provide a critical assessment of the validity and reliability of survey questions that purport to provide a proper assessment of whether Americans are receiving timely and appropriate cancer screenings. This paper documents the methods we are planning and using for this validity and reliability study.

### Motivation

In January 2020, the Centers for Disease Control and Prevention solicited a contract to assess the validity and reliability of cancer screening questions on cervical, colorectal, breast and lung cancer screening. There have been studies comparing survey data reporting on cancer screening behavior with medical records for the past three decades [15–27]. Table 1 contains various measures of accuracy from these studies.

**Table 1:** Summary of studies estimating validity of self-report cancer screening questions.

Cancer Screening Type*	Study	Year(s)	Population Studied	Validation and gold standard	Concordance	Se [CI] / Sp [CI]	PPV [CI] / NPV [CI]	Reports to Records Ratio
**Colorectal Cancer Screening**								
Colonoscopy	Vernon (2008) [15]	9/2005-12/2006	English-speaking men and women between 51–74 years of age and receive care at least five years at KSC. [Kelsey-Sebold Clinic, Houston, TX]	Self-report of adherence to colorectal cancer screening guidelines compared to administrative database and medical records. This study used the National Cancer Institute Health Information National Trends survey.	.91	.91/.91		1.15
FOBT	Vernon (2008) [15]				.85	.82/.86		1.57
Sigmoidoscopy	Vernon (2008) [15]				.85	.76/.89		1.1
BE	Vernon (2008) [15]				.92	.56/.97		0.82
Colonoscopy	Katz (2022) [22]	October 2016-June 2019	English speaking women who between 50–74 years old and residing in a rural county of Indiana or Ohio.	Self-report of up-to-date cancer screenings compared to medical record.	.90 (.89-.92)	.95 (.93-.96)/.85 (.82-.88)	0.88 (0.86–0.90)/ 0.94 (0.92–0.95)	
FIT/FOBT	Katz (2022) [22]				.94 (.92-.95)	.58 (.47-.69)/,96 (.95-.97)	.45 (.36-.55)/.97 (.97-.98)	
Colonoscopy	Reiter (2013) [21]	9/2009-4/2010	Appalachian Ohio residents ages 51–75.	Self-report of CRC data and agreement with recommended American Cancer Society CRC guidelines compared to medical record data.	0.80 (0.77–0.83)	0.96 (0.93–0.98)/ 0.65 (0.60–0.70)	0.71 (0.67–0.75)/ 0.95 (0.91–0.97)	1.35 (1.27–1.43)
FOBT	Reiter (2013) [21]				0.90 (0.88–0.93)	0.32 (0.17–0.51)/ 0.93 (0.91–0.95)	0.17 (0.09–0.29)/ 0.97 (0.95–0.98)	1.87 (1.27–2.75)
Flexible Sigmoidoscopy	Reiter (2013) [21]				0.96 (0.94–0.97)	0.17 (0.00–0.64)/ 0.96 (0.95–0.98)	0.04 (0.00–0.19)/ 0.99 (0.98–1.00)	4.50 (1.91–10.61)
Colonoscopy	Dodou (2014) [23]		Meta analysis	Medical records used in studies to compare accuracy of self-report.		0.425 (0.169)/ −0.311 (0.325)		
Sigmoidoscopy	Dodou (2014) [23]					0.849 (<0.001)/ −0.816 (<0.001)		
FOBT	Lofters (2015) [25]	(2001–2007)	Ontario, Canada residents eligible for breast, cervical, and colorectal cancer screening.	Validation of self-report of up-to-date cancer screenings compared to administrative data. This study used questions from the Canadian Community Health Survey.		.77(.757-.79)/.89(.892-.903)	.62[.60-.64]/.95(.94-.95)	1.25(1.19–1.31)
FOBT	Gordon (1993) [20]	N/A	Kaiser Foundation Health Plan members aged 40–74 living in Northern California region for 5 years prior to study.	Validation of self-reported interval since last screening within past 2 years compared to medical records.	78.9	98.1/70.6	8.2/29.3	
Sigmoidoscopy	Gordon (1993) [20]				86.8	79.4/87.5	20.6/12.5	
**Breast Cancer Screening**								
Mammography	Katz (2022) [22]				.91 (.90-.93)	.96 (.95-.97)/.78 (.73-.82)	.93 (.91-.94)/.87 (.83-.9)	
Mammography	Nandy (2016) [24]		Korean American women eligible for mammography	Accuracy of self-report of most recent mammogram date compared to medical records.	.80			
Mammography	Lofters (2015) [25]					.96(.963-.97)/.64(.63-.66)	.821(.81-.828)/.92(.91-.93)	1.18(1.16–1.20)
Mammography	Anderson (2019)		Meta analysis	Accuracy of self-report compared to medical records.	.82(.79-.86)	.96(.95-.98)/.61(.53-.69)	.80(.79-.81)/.86(.85-.87)	
Mammography	Gordon (1993) [20]				83.7	98/50.6	2.0/42.0	
**Cervical Cancer Screening**								
Pap/HPV	Katz (2022) [22]				.82 (.80-.84)	.94 (.92-.95)/.69 (.65-.72)	.78 (.75-.81)/.90 (.87-.93)	
Pap test	Lofters (2015) [25]					.97(.963-.967)/.50(.49-.51)	.83(.82-.83)/.85(.84-.86)	1.17(1.16–1.18)
Pap test	Stewart (2016) [27]	11/2007-07/2009	Aboriginal and Torres Islander residents, ages 18–69 receiving care at an Aboriginal Community Controlled Health Service organization	Accuracy of self-report of most recent pap test within recommended guidelines compared to pathology records.		.90(.55-.98)/.45(.38-.52)	.065(.03-.12)/.99(.95–1)	
Pap test	Anderson (2019)				.81(.77-.84)	.96(.94-.97)/.48(.41-.56)	.84(.83-.86)/.83(.82-.84)	
Pap test	Gordon (1993) [20]				78.4	97.2/34.9	2.8/65.1	

*-Cancer Screening Test Abbreviations: FOBT-fecal occult blood test; FIT-fecal immunochemical test; BE-barium enema test; Pap-Papanicolaou Smear Test; HPV-human papilloma virus test.

For example, soon after the NHIS included cancer screening questions, Gordon, Hiatt, and Lampert performed an interview study of 431 women and 348 men comparing survey responses to medical records for six cancer screening tests, three identical to ours: Pap smear, mammography, and fecal occult blood test; and three tests not in much use today: clinical breast examination, sigmoidoscopy, and digital rectal examination. Concordance as well as measures of sensitivity between self-report and medical records appeared quite high. For concordance, the estimates were: Pap 78%, FOBT 79%, Mammography 84%, and Sigmoidoscopy 87% [20].

In 2008, Vernon et al. performed a reliability and validity study of the NHIS colorectal cancer screening questions among 857 men and women ages 51–74 [15]. They found high levels of concordance at or above 0.85 with medical records for questions about fecal occult blood testing, sigmoidoscopy, colonoscopy, and barium enema (BE). This study also looked at reliability and validity by survey mode and demonstrated no differences between phone, mail, and face-to-face survey modalities. This study was conducted before newer tests such as Cologuard were used for colorectal cancer screening, and it is unknown whether screening knowledge or behavior is different with this highly marketed test [35]. More recently, studies by Reiter et al. (2013), Katz et al., (2022), Dodou, et al., (2015) and Nandy, et al., (2016) [21–24] show similar patterns of concordance between self-report and EMR data (Table 1); however, none of these studies used NHIS questions. While there are several comparisons of patient self-report corresponding closely to data found in matched medical records for colonoscopy, there are notable differences in these two data sources for other tests such as FOBT, FIT and BE.

At the time that the report by Vernon emerged, Rauscher and colleagues [28] performed a meta-analysis of self-reported cancer screening histories. While they concluded that accuracy figures were generally high, particularly sensitivity, they also concluded, “When estimates of self-report accuracy from this meta-analysis were applied to cancer-screening prevalence estimates from the National Health Interview Survey, results suggested that prevalence estimates are artificially increased and disparities in prevalence are artificially decreased by inaccurate self-reports [28].”

In a similar systematic review, though in this case focused specifically on mammography from 1990 through 2017, Levine, et al. (2019) state that their “review of the totality of published evidence suggests a lack of validity of self-reports of mammography [29].”

In aggregate, these studies do not show a consistent pattern of high correlation between survey results and electronic medical records. While the agreement measures tend to be higher for both mammography and colonoscopy, there is generally lower agreement for tests such as fecal occult blood tests, FIT tests, and to a lesser extent Pap smears and HPV testing. This degree of variability in various concordance measures remains one reason to continue to evaluate the NHIS battery of questions and determine their ability as indicators for our US screening adherence rates.

## Methods

### Ethical approval

The recruitment, consent, and study procedures were reviewed and approved by the University of Washington Institutional Review Board (STUDY 12071). KPWA, and MHS sites ceded human subjects review to the University of Washington. Henry Ford Health obtained IRB approval from Henry Ford Health Institutional Review Board (STUDY 16261). To obtain informed consent, study research staff working at each site contact the potential participant by sending an invitation letter with a description of the research project that explains key eligibility requirements and logistical aspects of study participation to their mailing address listed in the site’s EHR. Participants completing the survey over the phone with research staff or via web are explained that their participation on the study is voluntary and their decision to participate will not impact or change the benefits or medical care they receive. Research staff ask the phone-based participants to confirm they have read the study information letter and to provide verbal consent to participate. Participants completing the web version of the survey confirm having read the study information letter and provide digital consent. Verbal and digital consent is documented for each participant in our databases.

### Setting

Three health systems were initially included to achieve diverse recruitment in the Pacific Northwest, and a fourth in Michigan was later added to achieve further increase diversity in our target population. These sites include the University of Washington (UW), Kaiser Permanente Washington (KPWA), MultiCare Health System (MHS), and Henry Ford Health (HFH). These systems have primary care populations with comprehensive EMRs and cancer registry data. The three PNW health systems are recruiting people eligible for breast, colorectal, cervical, and lung cancer screening, whereas HFH is only recruiting people eligible for lung cancer screening. However, if HFH participants are eligible for additional cancer screening, they could still complete those screening questions.

### Design

In order to test whether mode of survey administration affected cancer screening question validity, we are embedding the surveys within a randomized trial of phone- vs. web-based survey questions about breast, colorectal, cervical and lung cancer screening compared to gold-standard data from EMRs. To measure the reliability of the cancer screening questions, we simultaneously randomize participants to receive a follow-up survey either 1- or 3-months after initial survey completion.

### Participants

Patients from KPWA are eligible to participate if they have at least five years of continuous enrollment before the date of their data pull. To recruit a similar population at the other sites, which do not have a defined enrolled population, we require evidence of interaction with the health system prior to the date of their data pull. Patients from UW, MHS, and HFH are eligible to participate if they have evidence of an outpatient visit or a hospitalization in at least three of the five years prior to the date of their data pull. While we focus our sample selection at Henry Ford on those eligible for lung cancer screening, we ask respondents about cervical, breast, and colorectal cancer screening if they are eligible, in order not to stigmatize the potential respondents who are current or former smokers.

Participants were initially eligible to take the survey if they met age and sex-based cancer screening recommendations (based on biological sex from health plan data) and smoking history criteria for lung cancer screening from the USPSTF recommendations as shown in Table 2. In 2021, the USPSTF updated their cancer screening guidelines for colorectal and lung cancer; however, our study chose not to adopt the updated recommendations as sites did not immediately adopt these changes (see notes in Table 2) [13, 14, 30–32]. Potential participants are excluded from the study if they do not fit the age criteria, do not speak English or Spanish, do not have a 30 pack-year smoking history (for the lung screening questions), and/or had hysterectomy or colectomy (for the cervical and colorectal questions, respectively). Potential participants who had a personal history of cancer are also excluded, as we found our questions could be burdensome to those individuals, and the appropriate screening routine for them may be different or more intense.

**Table 2:** Current USPSTF screening recommendations, study definitions, and eligibility criteria.

Site	Current USPSTF screening recommendations	Up-to-date screening definition	Inclusion criteria by recruitment categories	Study population exclusions	Notes
Breast	Individual decision to start screening every 2 years for women 40–49 years Screening every 2 years for women aged 50–74 years	Mammogram in 2 years before data pull	Breast, cervical, and colorectal: females ages 50–65 Breast and colorectal: females ages 66–74 Breast and cervical: females ages 40–49	Prior cancer diagnosis Do not speak English or Spanish*	
Cervical	Cytology screening every 3 years in people with a cervix aged 21–29 Cytology screening alone every 3 years for people with a cervix aged 30–65 OR HPV testing every 5 years for people with a cervix aged 30–65 OR Co-testing (cytology plus HPV testing) every 5 years for people with a cervix aged 30–65	Cytology alone in 3 years before data pull OR HPV testing (with or without cytology) in 5 years before data pull	Cervical-only: females ages 21–39	Prior cancer diagnosis Prior hysterectomy Did not speak English or Spanish*	
Colorectal	FOBT or FIT test every year for adults aged 45–75 years OR FIT-DNA test every 3 years for adults aged 45–75 years OR Sigmoidoscopy every 5 years for adults aged 45–75 years OR Sigmoidoscopy every 10 years plus annual FIT for adults aged 45–75 years OR CT colonography every 5 years for adults aged 45–75 years OR Colonoscopy every 10 years for adults aged 45–75 years	FOBT or FIT in 1 year before data pull OR Sigmoidoscopy or colonoscopy within 5 years before data pull	Colorectal-only: males and females ages 50–75	Prior cancer diagnosis Prior colectomy Adults ages 45–49 Do not speak English or Spanish*	We do not include adults ages 45–49 despite USPSTF screening recommendations because health care systems are in the process of adopting this new recommendation during the study period. We will not look back for 10 years for colonoscopy history because this would limit our eligible sample to a very select population.
Lung	LDCT every year in adults aged 50–80 years who have a 20 pack-year smoking history AND currently smoke or quit within the past 15 years	LDCT in 1 year before data pull	Lung-only: males and females ages 55–75 with 30+ pack-year smoking history	Prior cancer diagnosis <30 pack-year smoking history Do not speak English or Spanish*	We do not include adults with a 20–29 pack-year history of smoking or those 55 and younger) despite USPSTF screening recommendations because health care systems were in the process of adopting this new recommendation during the study period.

Abbreviations: HPV (human papillomavirus), FOBT (fecal occult blood test), FIT (fecal immunochemical test), CT (computed tomography), LDCT (low dose computed tomography)

*Spanish surveys added 1 year after starting data collection at UW and MHS sites.

### Stratification and randomization

We extract data on prior cancer screening from EMRs using Common Procedural Terminology (CPT) codes, International Classification of Disease (ICD) codes, and home-grown codes from each health plan. Participants are stratified by screening status into three groups based on EMR data: up to date with screening based on USPSTF recommendations, screened in the past five years but not up to date with their screening, and eligible but not screened in the past five years. Participants are stratified by race and ethnicity with the goal of recruiting at least 25% Non-Hispanic Black participants and 25% Hispanic participants for the study population. If not enough available participants are available to recruit from a given stratum, we select additional participants from other strata. Random samples of eligible participants from each study site are sent to the UW Data Coordinating Center (UW DCC) for stratified randomization for initial survey modality (phone vs. web) and subsequent survey timing (1 vs 3 months) on a regular basis at KPWA, UW, and MHS. Due to administrative requirements at HFH, patients at this site ware randomly assigned to their survey modality but not their follow-up time. We select HFH patients for participation in two waves. Those selected in May 2023 were assigned to 3-month follow-up, whereas those selected in August 2023 were assigned to 1-month follow-up. To maximize study efficiency, we select people who are eligible for more than one screening exam based on EMR information. For example, we combine cervical and breast cancer screening questions for women ages 40–49 and breast, cervical, and colorectal cancer screening for women aged 50–65 (Table 2).

### Sample size

**Fig 1:**
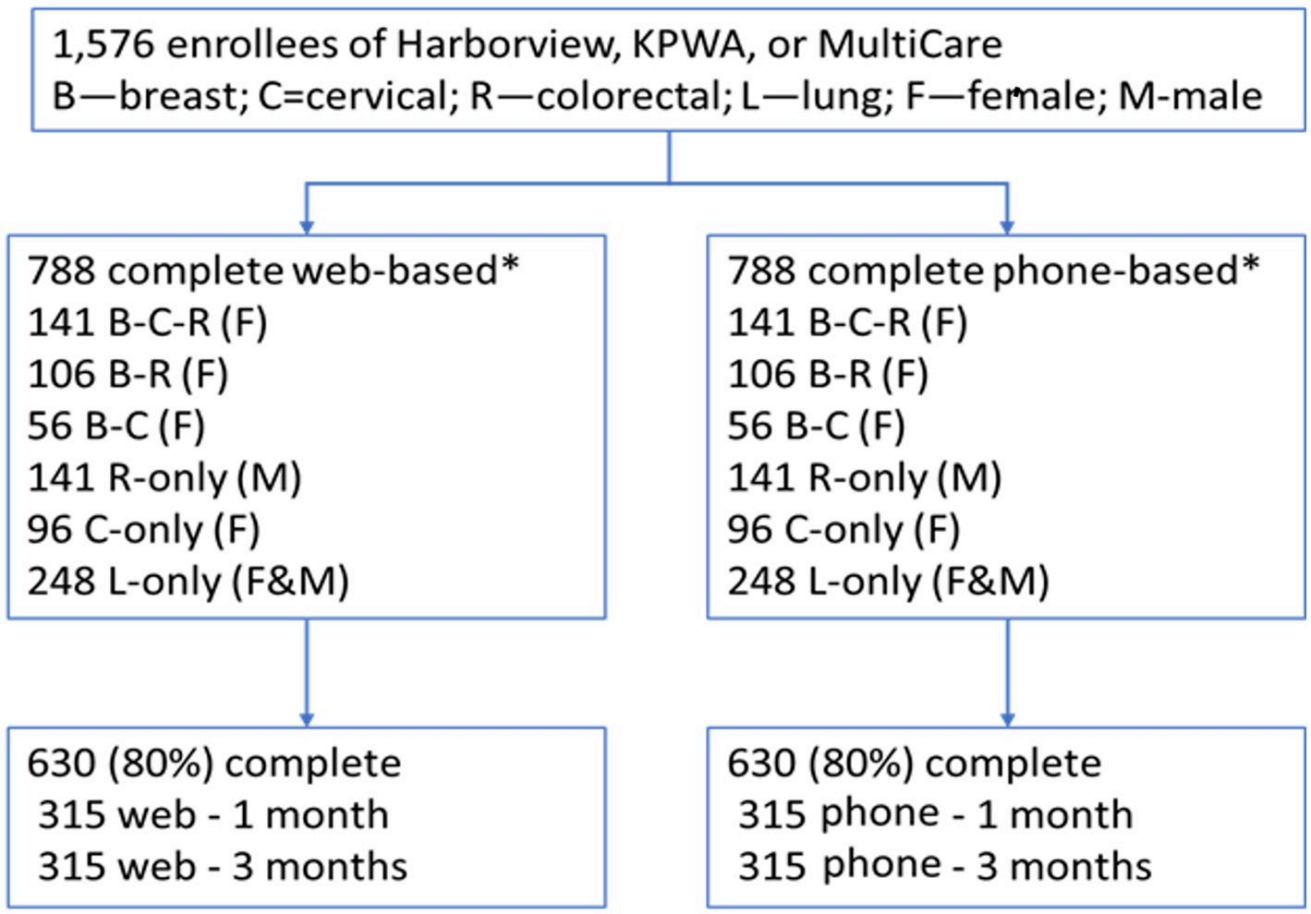
Design for overall and interview mode sub-study.

A minimum of 500 gold-standard positive and negative participants for each cancer type are selected to obtain confidence interval width of <0.1 for sensitivities and specificities >0.80 across all survey modalities. A total study sample size of at least 1,576 individuals will contribute information to validity analyses of cancer screening history (Fig 1). For subgroups representing 20% (n = 100) of each cancer screening cohort, highly concordant screening tests (>0.9) and tests with good concordance (>0.8) will have 95% confidence interval widths of <0.12 and <0.16 respectively. We estimate that 1,260 (80%) participants would need to be included for the assessment of test-retest reliability across all screening modalities, resulting in >0.80 power to rule out Kappa coefficients smaller than 0.61 if the true test-retest agreement is greater than 70%.

### Instruments/Measures

We conducted systematic cognitive interviewing of colorectal, breast, cervical, and lung cancer screening questions from the 2021 National Health Interview Survey in both English and Spanish to ensure these questions were well-understood by our populations (S1 Appendix). We performed one sub study on cervical cancer at the UW and University of Texas Southwestern and this was analyzed separately [33]. Both web- and phone-based validity surveys included eligibility questions, demographic questions, and cancer screening questions. This led to several important changes to the NHIS questions. Through cognitive testing we learned that some participants had challenges determining the main reason for their most recent exam. The question was initially a single question that included multiple options that participants could choose as the main reason for their most recent exam, such as “routine,” “follow-up to a recent exam,” or “because of a problem.” When given the option, respondents generally did not want to choose only one answer to this question. To improve the ability to answer the intent of this single question, we created a three-question series where we separated each reason into an individual question with dichotomized yes/no options. Additionally, we found that exams with similar procedures were confusing for some participants; therefore, we added additional details to help improve the clarity of the exams. For example, some participants in the cognitive surveys had challenges distinguishing between colonoscopy and sigmoidoscopy exams; therefore, we separated the questions about these exams and added more details about the exam procedures. Similarly, we added explanatory information for the Cologuard test, which participants had challenges distinguishing from the blood stool FIT test. We included more specifics to explain the differences in sample collection process of both exams. We performed a second round of cognitive testing and found our modification of the questions improved participants’ understanding of questions included in the survey.

### Recruitment and data collection

Each site is responsible for the recruitment and interviewing of participants from their healthcare system. As described above, each site regularly sends samples of eligible participants to the UW DCC for stratified selection and randomization. The UW DCC returns a list of randomized study participants back to each site. Staff from each site mails recruitment and study information letters to these potential participants. The recruitment letters include information on their assignment to either the phone or web survey. If assigned to the web survey, the recruitment letters include a survey link and QR code along with a unique study ID in two parts (ID and PIN). If assigned to the phone survey, potential participants are notified in the letter that a member of the study team would be calling them to conduct their interviews. Trained interviewers call participants one week after the letters are sent. Participants are called up to 5 times if they do not complete the survey online or are unavailable by phone. For each group, we offer the option to complete the survey via an alternate modality for participants who ask for this option (i.e., phone participants are emailed a web link if they preferred to complete it online and web participants are offered the opportunity to do the survey over the phone).

Invited participants complete eligibility questions and if eligible, respond to the cancer screening questions. The cancer screening survey questions include questions on whether the participant had received cancer screening exams, when they received their most recent cancer screening exam, the type of exam they received, and the main reason for their most recent screening exam. Participants are asked the most important reason for not being screened if the participant has not recently received a cancer screening or is not up to date with the specific type of cancer screening.

For reliability, staff from each site are mailed a second letter to participants either 1- or 3-month(s) after they complete the initial survey, if they complete at least one cancer screening question on the initial survey. Trained interviewers conduct follow-up in the same manner as the initial survey. Participants are asked to complete the same survey questions they complete in the initial survey, using the same modality (phone or web) they use for their initial survey. We send reminders to improve the response rate. We mail participants cash incentives for completing the validity ($10) and reliability ($15) surveys.

### Validation

To test the validity of the cancer screening questions, we will compare participants’ survey responses to gold standard EMR data. Not all questions on the survey can be validated. For example, we cannot validate questions about whether the doctor explained the exams at the visit or about the cost of procedures. In these cases, either the information is not recorded, or not available in EMR data.

To make these comparisons, we will obtain EMR data (e.g., utilization data on procedure and diagnosis codes) before each person’s survey. UW, KPWA, MHS, and HFH have extensive automated healthcare utilization data, which include enrollment (for KPWA) and demographics, diagnoses, procedures, outside claims, and cancer diagnoses. Electronic data are available for the entire study period and are housed in enterprise data warehouses at each site where they are readily available to programmers. Within these health systems, we can accurately identify whether each person sad any prior exams in the past five years with their results, dates, types of exams, and indication. We have used EMR data in many previous studies at KPWA and similar institutions to identify cancer screening tests [34–36]. As an insurer, KPWA also obtains claims for procedures that occur at non-KPWA facilities. For the purposes of assessing validity, these data will be considered the gold standard with which to compare self-reported responses on each screening questionnaire. The focus of the questions for validation are the ones for each cancer site that directly measure the outcomes for the Healthy People objectives. Our analysis of questions and their validity will focus on what the specific screening examination was, when was the last exam, and the main reason for the exam.

### Statistical analyses

#### Primary aims and analyses on validity

For each cancer site, as well as for groups of related questions, we will construct a misclassification matrix for each question from the screening history questionnaires, by cancer site and question group (related questions) (Table 3). All variables will be dichotomous (prior exam in the last 5 years [yes/no], exam type [e.g., co-test, primary HPV, Pap; colonoscopy yes/no], indication [screening yes/no]) or can be dichotomized (screening interval [e.g., </> recommended screening interval]). To correctly characterize agreement, we will need to ensure the tests from the medical records are classified as screening or diagnostic. In some cases, standard codes are available. In other cases, the time interval between exams from the EMR may be helpful in evaluating whether tests received were intended for screening or diagnostic evaluation. We will adopt the approach taken by Vernon, et al. (2008), and focus on measures of concordance, including sensitivity, specificity, reports to records ratio, positive and negative predictive values (PPV and NPV), and Cohen’s Kappa (level of agreement) [15]. Specifically, we will evaluate:

- Sensitivity (probability of self-reported screening test when a test was received)
- Specificity (probability of no self-reported screening test when no test was received)
- Overall concordance (percentage agreement)
- Reports to records ratio (percentage of self-reported screening tests divided by percentage of records with a screening test; a ratio >1.0 indicates over-reporting, and <1.0 indicates under-reporting)
- PPV: the proportion of truly having been screened when self-reported screening.
- NPV: the proportion of no screening test when no self-reported screening.

**Table 3:** Misclassification matrix for dichotomous (or derived dichotomous) question outcomes.

	Electronic Health Record Gold Standard		
Self-Reported Survey	Outcome present	Outcome absent	
Outcome identified	A (true positives)	B (false positives)	PPV = A/(A+B)
Outcome not identified	C (false negatives)	D (true negatives)	NPV = D/(C+D)
	Sensitivity = A/(A+C)	Specificity = D/(B+D)	Concordance = (A+D)/(A+B+C+D); Reports to records = (A+B)/(A+C)

For sensitivity, specificity, PPV, NPV, and concordance we will calculate 95% confidence intervals based upon the binomial distribution. Sensitivity and specificity indicate the effectiveness of a test (here the NHIS survey result) with respect to a trusted “outside” referent, while PPV and NPV indicate the effectiveness of a test (the NHIS survey result) for categorizing people as having or not having a target condition/screening [37].

We will also calculate the report-to-records ratio, the ratio of participants reporting a test (true positives plus false positives) divided by the percentage of tests in the record (true positives plus false negatives). The report-to-records ratio is a measure of net bias in test reporting, where values >1.0 denote overreporting and values <1.0 denote underreporting [38]. All measures will be calculated for each cancer screening test type, as well as by mode of survey administration. For reports to records ratio, 95% confidence intervals will be generated using bootstrap resampling [39].

In our analysis, we will compare our accuracy measures for those completing the survey by web vs. phone. Because survey modality (phone vs. web) was randomly allocated, and we allow participants to change their mode of completion when we contact them, we will perform both an intent-to-treat analysis as well as a separate analysis by modality used.

For responses to the question ‘when did you have your most recent test to check for [cervical/breast/colorectal/lung] cancer’, we hypothesize response accuracy will vary based on the length of time since the last screening exam. We will present the proportion of patients who answer ‘yes’ to having had screening stratified by ranges of time between the survey date and time of last screening exam, including a stratum for those who have not had this exam within the study reporting period of 5 years. In addition, we will present the proportion of patients who had screening exams, within these ranges of time, among those who answer ’yes’ or ’no’ to each question. All analyses will be stratified by cancer site.

#### Secondary aims and sensitivity analyses on validity

Whereas our primary analysis will include all persons interviewed regardless of modality, in these secondary analyses, we will describe and compare validity between web and phone-based survey modalities. We will perform analyses for each cancer site and control for demographic and clinical factors, such as age, sex, race/ethnicity, health system, and web vs. phone completion of the questionnaire. If there are statistically significant differences between web and phone-based surveys with respect to agreement measures, we will consider statistical adjustments for analyses or presentation by separate modality where we have sufficient sample sizes.

We will also conduct a per-protocol analysis (including only those surveyed with the assigned modality) and an as-surveyed analysis (including all respondents according to the completed survey modality). A Wald-based test will be used to assess the statistical evidence for differences across subgroups (*p*<0.05, with no adjustment for multiple testing).

We will further use a multivariable logistic regression model of participants’ reported screening status (not answering “yes/no”, but “correct/incorrect” answer) as a function of actual screening status (i.e., whether and how recently they have been screened) to estimate the degradation of recall over time. We will perform analyses for each cancer site and control for demographic and clinical factors, such as age, sex, race/ethnicity, health system, and web vs. phone completion of the questionnaire. Heterogeneity in the relationship between actual and reported screening status will be accounted for by modeling the interactions between actual screening status and the factors listed above on the outcome of reported screening status.

#### Reliability analyses

In contrast to validity, reliability analyses have a relatively simpler task in answering the following question: are the reports of screening behavior on an initial survey congruent with a follow-up survey regardless of the accuracy of the behavior with respect to EMR data?

Test-retest reliability, the reproducibility of a measure [40], will be assessed for each type of cancer screening. We will code a participant’s responses as consistent if the time interval between the survey date and the self-reported month and year was within guidelines on both the validation and reliability surveys, or if no test within guidelines was reported on both surveys. Patients with a screening test documented in the EMR for a given cancer site between completion of the validity and reliability surveys, or who were up to date with screening at the time of the validity survey but not at the time of the reliability survey due to the passage of time, will be excluded from reliability analyses due to the possibility that their true cancer screening status may have changed.By cancer type and survey modality, we will use Cohen’s Kappa [41] statistic to assess repeatability while correcting for chance agreement between survey administration time points. Kappa coefficients >0.80 are often used to indicate *excellent* agreement while *Kappa* coefficients between 0.61 and 0.80 indicate *substantial* agreement [42]. For participants whose screening status changes during the period between the baseline and follow-up surveys (e.g., due to recently being screened), response concordance will be measured against the updated screening status. As with the validity analyses, we will also analyze reliability by the time of the last recorded screening examination in groups, such as within 6 months, 6 months to less than a year, etc., as well as by questionnaire modality (web vs. phone). That is, we will see if the reliability coefficients for an answer to recent screening depend on the length of time to the last screening examination.

#### Missing data

Missing data due to non-response on both survey waves will be investigated in-depth to look for potential associations with participant demographic factors or with responses to the previous survey. We will use a multiple imputation approach as a sensitivity analysis to examine the extent to which missing data may influence the observed reliability results.

## Discussion

Previous studies attempting to validate cancer screening questions have demonstrated a variety of findings, from generally high levels of concordance (exceeding 90%) for mammography and either flexible sigmoidoscopy or colonoscopy, to very mixed or poor concordance for other cancer screening tests. The recency of recommendations for lung cancer screening with low-dose computed tomography means no evidence currently exists about self-report to medical record validation. An important observation about many of these validation studies is that they have generally shown a tendency for self-report to overestimate screening adherence. That is of specific importance to those measuring national adherence as it would give those programs a systematically biased and false estimate of success. Figuring out the degree of such overreporting will be one characteristic of our analyses.

These previous validation studies have also demonstrated that obtaining accurate cancer screening history information requires asking people more than just a yes/no question. Surveys must ask about any prior cancer-related results, test type, interval, and indication, as these constructs are interrelated. In cervical cancer screening, for example, the length of the routine screening interval varies by screening modality (e.g., three years for a Pap test alone versus five years for Pap and HPV co-testing) (Table 2). We must also consider how question responses are influenced by demographic and socioeconomic factors—several of which have been shown to affect validity of self-reported questionnaires on screening history [25, 27].

In many countries, organized programs for cancer screening exist, and these programs, along with population registries, have the advantage over the US of being able to calculate compliance with screening recommendations. It might be of considerable value to mount a cooperative international effort, perhaps under the International Cancer Screening Network (ICSN), to conduct surveys to look for reasons for non-compliance and assess ability to recall screening behavior. We are unaware of any such current effort.

### Limitations

We recognize that EMR data have flaws. For example, coding is sometimes done in billing departments, and thus, the indication for use of a particular test may have inaccuracies. In addition, some tests, such as colonoscopy, can present challenges, such as the finding of a pre-cancerous polyp changing what had been indicated as a screening examination to one that may be now coded as a diagnostic examination. Missing tests, such as those that may be sent to a patient’s home for stool sampling, will also occur. However, they represent the strongest evidence with which to compare self-reported data. We recognize the limitations associated with this electronic approach but acknowledge that this method is similar to what health systems use to calculate HEDIS performance [43].

Qualitative studies are essential for understanding the reasons for not participating in screening, which are not possible to verify with our study approach. As an additional potential limitation, we note that the population included in the survey may not capture information about people of interest where information would be helpful to plan interventions. There might be other risk factors that prevent people from utilizing screening tests, such as lower health literacy, which is hard to measure, yet could interfere with patients’ study enrollment response. Hiatt et al. (2002) note sociodemographic correlates, health care system correlates, knowledge/behavioral/attitudinal correlates, health status/health profile correlates could have an impact on agreement indices, none of which we will be able to evaluate [10]. Although we are able to conduct interviews in Spanish, there will be too few in this study to perform separate analyses to determine the influence of language on screening understanding and recall. Other languages will not be attempted, but the results of this study would continue to establish a current baseline on concordance to serve as a reference for other investigators. We are aware that the prevalence of cancer screening uptake in the population may impact sensitivity and specificity. Thus, comparing those indices across cancer screening modalities with substantial uptake discrepancies may cause challenges.

It is also likely that those who have never been screened may well be less likely to join a study like ours. This would result in an incorrect estimate in screening from surveys that we would not specifically address with these type of validity studies. However, we can estimate response rates by screening status to determine whether or not this is the case in our study.

## Conclusions

We think that our design and methodology have unique features, such as long period of lookback for screening compliance, an embedded randomized trial comparing phone to web interviewing, and multiple health systems that span a range of populations, that can assist in verifying the degree of misreporting, particularly with respect to telescoping. We hope to quantify the degree of misestimation and recommend new approaches to measuring validity and reliability of these cancer screening questions.

## Supporting information

S1 Appendix Questionnaires from the NHIS. (PDF)

S2 Appendix Decisions related to specific cancer screening exams. (PDF)

S3 Appendix Questions that cannot be validated. (PDF)
