# Supplementary material for: Protocol to measure validity and reliability of colorectal, breast, cervical and lung cancer screening questions from the 2021 National Health Interview Survey: Methodology and design
Source: PLoS One. 2024 Mar 4;19(3):e0297773. doi: 10.1371/journal.pone.0297773 (PMC10911603; doi:10.1371/journal.pone.0297773)
Supplement: S1 Appendix — (PDF) [file pone.0297773.s001.pdf]

NHIS Revised Cancer Screening Questions

Cervical Cancer Screening Questions

CERVICEV1_A

1. There are two different kinds of tests to check for cervical cancer. One is a Pap smear or Pap test and the other is the HPV or Human Papillomavirus test. These are routine tests for women in which a doctor or other health professional takes a sample from the cervix with a swab or brush and sends it to the lab. These tests can be done alone, or at the same time.
   Have you ever had a test to check for cervical cancer?
   1) Yes
   2) No
   3) Refused
   4) Don't Know

Skip Pattern:	
<1>	[go to CERVICWHEN_A]
<2>	[go to CERVICNOT1_A]
<3, 4>	[go to HYSTEV1_A]

CERVICWHEN_A

2. When did you have your MOST RECENT test to check for cervical cancer?

   1) Within the past year (anytime less than 12 months ago)
   2) Within the past 2 years (1 year but less than 2 years ago)
   3) Within the past 3 years (2 years but less than 3 years ago)
   4) Within the past 5 years (3 years but less than 5 years ago)
   5) Within the past 10 years (5 years but less than 10 year ago)
   6) 10 years ago or more
   7) Refused
   8) Don't Know

Skip Pattern:	
<1-8>	[go to CERREASON1_A]

CERREASON1_A

Thinking about this last cervical cancer exam, was this because of a problem with your health?

1) Yes
2) No
3) Refused
4) Don’t Know

Skip Pattern:	
<1>	[go to PAPTEST_A]
<2-4>	[go to CERREASON1_B]

CERREASON1_B

Was this as a follow-up test of an earlier test or screening exam?

1) Yes
2) No
3) Refused
4) Don’t Know

Skip Pattern:	
<1>	[go to PAPTEST_A]
<2-4>	[go to CERREASON1_C]

CERREASON1_C

Was this last cervical cancer exam part of a routine exam or a screening exam?

1) Yes
2) No
3) Refused
4) Don’t Know

Skip Pattern:	
<1-4>	[go to PAPTEST_A]

PAPTEST_A

3. At your most recent cervical cancer screening, did you have a Pap test or Pap Smear?
   1) Yes
   2) No
   3) Refused
   4) Don't Know

Skip Pattern:	
<1-4>	[go to HPVTEST_A]

HPVTEST_A

4. At your most recent cervical cancer screening, did you have an HPV test? HPV stands for Human Papillomavirus.
   1) Yes
   2) No
   3) Refused
   4) Don't Know

<1,2, RF, DK>	[go to TELLCERVIC_A]

TELLCERVIC_A

5. At your MOST RECENT cervical cancer screening, did a doctor or other health professional tell you which type of test or tests you received?
    1) Yes
    2) No
    3) Refused
    4) Don't Know

Skip Pattern:	
<1, 2, RF, DK>	If CERVICWHEN_A IN (1,2,3,4) [go to CERVICRES_A]<br>if CERVICWHEN_A IN (5,6) [go to CERVICNOT1_A]<br>if CERVICWHEN_A IN (RF,DK) [go to HYSTEV1_A]

CERVICRES_A

6. In the PAST 5 years, did any of your cervical cancer screening tests require follow-up to check for cancer or precancerous cells?
    1) Yes
    2) No
    3) Refused
    4) Don't Know

Skip Pattern:	
<1, 2, RF, DK>	Go to HYSTEV1_A]

[If the participant has not had a cervical cancer test in the past five years]
CERVICNOT1_A

5) What is the main reason that you have not had a test to check for cervical cancer in the last five years?

1) No reason/never thought about it
2) Didn’t need it/didn’t know I needed this type of test
3) Doctor didn’t order it/didn’t say I needed it
4) Haven’t had any problems
5) Put it off/didn’t get around to it
6) Too expensive/no insurance/cost
7) Too painful, unpleasant, or embarrassing
8) Had hysterectomy
9) Don’t have a doctor
10) Had HPV vaccine
11) I am too old
12) I am too young
13) Other
14) Refused
15) Don’t know

<1-7,9-13, RF, DK>	Go to [go to HYSTEV1_A]

HYSTEV1_A

7. A hysterectomy is when the uterus or womb is removed, this is different from having your tubes tied. Have you had a hysterectomy?

    1) Yes
    2) No
    3) Refused
    4) Don't Know

Breast Cancer Screening Questions

MAMEV_A

1. Have you EVER HAD a mammogram? A mammogram is an x-ray taken only of the breast by a machine that presses against the breast.
    1) Yes
    2) No
    3) Refused
    4) Don't Know

Skip Pattern:	
<1>	Go to MAMWHEN_A
<2>	Go to MAMNOT1_A
<RF,DF>	Breast Cancer section complete

MAMWHEN_A

2. About how long has it been since your MOST RECENT mammogram?

    1) Within the past year (anytime less than 12 months ago)
    2) Within the past 2 years (1 year but less than 2 years ago)
    3) Within the past 3 years (2 years but less than 3 years ago)
    4) Within the past 5 years (3 years but less than 5 years ago)
    5) Within the past 10 years (5 years but less than 10 year ago)
    6) 10 years ago or more
    7) Refused
    8) Don't Know

Skip Pattern:	
<1-6,RF,DK>	[Go to MAMPAY_A]

MAMPAY

3. Did you pay none, part, or all of the cost for your most recent mammogram?

1) None of the cost
2) Part of the cost
3) All of the cost
4) Refused
5) Don't Know

Skip Pattern:	
<1-3,RF,DK>	Go to MAMREASON_A

Mamreason_A

4. Thinking about this last mammogram exam, was this because of a problem with your health?
    1) Yes
    2) No
    3) Refused
    4) Don't Know

Skip Pattern:	
<1>	Go to MAMAGE1ST1_A
<2-4>	Go to Mamreason_B

Mamreason_B

5. Was this as a follow-up test of an earlier test or screening exam?

    1) Yes
    2) No
    3) Refused
    4) Don't Know

Skip Pattern:	
<1>	Go to MAMAGE1st1_A
<2-4>	Go to MAMREASON_C

Mamreason_C

6. Was this last mammogram exam part of a routine exam or a screening exam?
    1) Yes
    2) No
    3) Refused
    4) Don't Know

Skip Pattern:	
<1-4>	Go to MAMAGE1st1_A

MAMAGE1ST1_A

7. About how old were you when you had your first mammogram?

    1) Under 30 years
    2) 30-39
    3) 40-49
    4) 50-59

5) 60 – 74
6) 75 years old or older
7) Refused
8) Don't know

Skip Pattern:	
<1-7,RF,DK>	End of Breast Cancer Screening questions

If participant has not had a mammogram in the past two years:
MAMNOT1_A

8. What is the most important reason why you have not had a mammogram in the past two years?

1. Didn't need it/didn't know I needed this type of test
2. Doctor didn't order it/didn't say I needed it
3. Haven't had any problems
4. Put it off/didn't get around to it
5. Too expensive/no insurance/cost
6. Too painful, unpleasant, or embarrassing
7. Don't have a doctor
8. I am too old
9. I am too young
10. Other
11. Refused
12. Don't Know

Colorectal Cancer Screening Questions
COLORECTEV_A

1. These next questions are about colorectal cancer screening. Colonoscopy is an exam to check for colon cancer. Have you ever had this exam?
Colonoscopy (colon-OS-copy) is an exam in which a doctor inserts a tube into the rectum to look for polyps or cancer. In a colonoscopy, the doctor checks the entire colon, and you are given medication through a needle in your arm to make you sleepy, and told to have someone take you home.
    1) Yes
    2) No
    3) Refused
    4) Don't Know

Skip Pattern:	
<1>	Go to COLWHEN_A
<2-4>	Go to SIGMOID_EV

COLWHEN_A

2. About how long has it been since your MOST RECENT colonoscopy?

    1) Within the past year (anytime less than 12 months ago)

2) Within the past 2 years (1 year but less than 2 years ago)
3) Within the past 3 years (2 years but less than 3 years ago)
4) Within the past 5 years (3 years but less than 5 years ago)
5) Within the past 10 years (5 years but less than 10 year ago)
6) 10 years ago or more
7) Refused
8) Don't Know

Skip Pattern:	
<1-8>	Go to Colreason_A

COLREASON_A

3. Thinking about this last colonoscopy exam, was this because of a problem with your health?
    1) Yes
    2) No
    3) Refused
    4) Don't Know

Skip Pattern:	
<1>	Go to Colpay_A
<2-4>	Go to Colreason_B

COLREASON_B

4. Was this as a follow-up test of an earlier test or screening exam?
    1) Yes
    2) No
    3) Refused
    4) Don't Know

Skip Pattern:	
<1>	Go to Colpay_A
<2-4>	Go to Colreason_C

COLREASON_C

5. Was this last colonoscopy exam part of a routine exam or a screening exam?
    1) Yes
    2) No
    3) Refused
    4) Don't Know

Skip Pattern:	
<1-4>	Go to Colpay_A

COLPAY_A

6. Did you pay none, part, or all of the cost for your most recent colonoscopy?

    1) None of the cost

2) Part of the cost
3) All of the cost
4) Refused
5) Don't Know

Skip Pattern:	
<1-4>	Sigmoid_Ev

SIGMOID_EV

7. Sigmoidoscopy is an exam where a doctor inserts a tube into the rectum and checks only the lower part of the colon for polyps or cancer. You are fully awake during the procedure. Have you ever had a sigmoidoscopy?

1) Yes
2) No
3) Refused
4) Don't Know

SIG

Skip Pattern:	
<1>	Go to SIGMOID_WHEN
<2-4>	Go to CTCOLEV1_A

SIGMOID_WHEN

8. When was your MOST RECENT sigmoidoscopy?

1) Within the past year (anytime less than 12 months ago)
2) Within the past 2 years (1 year but less than 2 years ago)
3) Within the past 3 years (2 years but less than 3 years ago)
4) Within the past 5 years (3 years but less than 5 years ago)
5) Within the past 10 years (5 years but less than 10 year ago)
6) 10 years ago or more
7) Refused
8) Don't Know

Skip Pattern:	
<1-8>	Go to CTCOLEV1_A

CTCOLEV1_A

9. CT colonography, sometimes called virtual colonoscopy, is a new type of test that looks for cancer in the colon. Unlike regular colonoscopies, you do not need medication to make you sleepy during the test. In this new test, your colon is filled with air and you are moved through a donut-shaped X-ray machine as you lie on your back and then your stomach. Have you ever had a CT colonography or virtual colonoscopy?

1) Yes
2) No
3) Refused

4) Don't Know

Skip Pattern:	
<1>	Go to CTCOLWHEN1_A
<2-4>	Go to FITHEV1_A

CTCOLWHEN1_A

10. When was your most recent CT colonography or virtual colonoscopy?
    1) Within the past year (anytime less than 12 months ago)
    2) Within the past 2 years (1 year but less than 2 years ago)
    3) Within the past 3 years (2 years but less than 3 years ago)
    4) Within the past 5 years (3 years but less than 5 years ago)
    5) Within the past 10 years (5 years but less than 10 year ago)
    6) 10 years ago or more
    7) Refused
    8) Don't Know

Skip Pattern:	
<1-6,RF,DK>	[Go to FITHEV1_A]

FITHEV1_A

11. The following questions are about the blood stool or occult blood test, fecal immunochemical or FIT test. These are tests to determine whether you have blood in your stool or bowel movement and can be done at home using a kit. You use a stick or brush to obtain a small amount of stool at home and send it back to the doctor or lab.

    Have you ever had a blood stool or FIT test, using a HOME test kit?

    1) Yes
    2) No
    3) Refused
    4) Don't Know

Skip Pattern:	
<1-6,RF,DK>	[Go to FITHWHEN1_A]

FITHWHEN1_A

12. When was your most recent blood stool or FIT test, using a home test kit?

    1) Within the past year (anytime less than 12 months ago)
    2) Within the past 2 years (1 year but less than 2 years ago)
    3) Within the past 3 years (2 years but less than 3 years ago)
    4) Within the past 5 years (3 years but less than 5 years ago)
    5) Within the past 10 years (5 years but less than 10 year ago)
    6) 10 years ago or more
    7) Refused

Skip Pattern:

<1-6,RF,DK>	[Go to COLOGUARD1_A]

COLOGUARD1_A

13. Cologuard or FIT-DNA is a new type of stool test for colon cancer that checks for blood in your stool. The Cologuard test is shipped to your home in a box. In this test, you have a bowel movement in the sample container and add a bottle of liquid preservative to the container, then you send the container back to the lab.

    Have you ever had a Cologuard test?
    1) Yes
    2) No
    3) Refused
    4) Don't Know

Skip Pattern:	
<1>	FITCOLG1_A
<2-4>	[Go to COLPROB1_A]

FITCOLG1_A

14. Was the blood stool or FIT test you reported earlier conducted as part of a Cologuard test?
    1) Yes
    2) No
    3) Refused
    4) Don't Know

Skip Pattern:	
<1>	[go to CGUARDWHE1_A]
<2-4>	[Go to COLPROB1_A]

CGUARDWHE1_A

15. When did you have your most recent Cologuard test?
    1) Within the past year (anytime less than 12 months ago)
    2) Within the past 2 years (1 year but less than 2 years ago)
    3) Within the past 3 years (2 years but less than 3 years ago)
    4) Within the past 5 years (3 years but less than 5 years ago)
    5) Within the past 10 years (5 years but less than 10 year ago)
    6) 10 years ago or more
    7) Refused
    8) Don't Know

Skip Pattern:	
<1-8>	Go to COLPROB1A

COLPROB1_A

1) Yes
2) No
3) Refused
4) Don't Know

Skip Pattern:	
<1>	Go to COLTEST_A
<2-4>	End of Colorectal questions

COLTEST_A

17. Which tests to check for colon cancer did the doctor or other health professional recommend to you? Possible tests include stool blood or fecal occult blood or FIT test; Cologuard or FIT DNA test; sigmoidoscopy; colonoscopy; CT colonography or virtual colonoscopy; or other.
    1) Stool blood or fecal occult blood or FIT test
    2) Cologuard or FIT DNA test
    3) Sigmoidoscopy
    4) Colonoscopy
    5) CT colonography or virtual colonoscopy
    6) Other
    7) Refused
    8) Don't Know

Lung Cancer Screening Questions
CTSCANEV_A

1. The following questions are about CT scans. During this test, you lie down on your back and are moved through an open, donut-shaped x-ray machine.
   Have you EVER HAD a CT scan?
    1) Yes
    2) No
    3) Refused
    4) Don't Know

Skip Pattern:	
<1>	Go to CTSCANCHST_A]
<2-4>	End of lung cancer screening survey

CTSCANCHST_A

2. Were any of the CT scans of your chest area?
    1) Yes
    2) No
    3) Refused
    4) Don't Know

Skip Pattern:	
<1, DK>	Go to CTLNGCAN_A
<2, RF>	End of lung cancer screening survey

CTLNGCAN_A

3. The next question is only about CT scans to check or screen for lung cancer, sometimes called low-dose CT scans. During a low-dose CT scan, you lie on your back on a long table as it slides through the center of a large machine that takes images of your lungs. This type of CT scan uses no dyes, no injections, and requires nothing to be swallowed by mouth.

Were any of the CT scans of your chest area done mainly to check or screen for lung cancer?

1) Yes
2) No
3) Refused
4) Don't Know

Skip Pattern:	
<1, DK>	Go to CTLNGWHEN_A
<2, RF>	End of lung cancer screening survey

CTLNGWHEN_A

4. When did you have your MOST RECENT CT scan of your chest area done mainly to check or screen for lung cancer?
   1) Within the past year (anytime less than 12 months ago)
   2) Within the past 2 years (1 year but less than 2 years ago)
   3) Within the past 3 years (2 years but less than 3 years ago)
   4) Within the past 5 years (3 years but less than 5 years ago)
   5) Within the past 10 years (5 years but less than 10 year ago)
   6) 10 years ago or more
   7) Refused
   8) Don't Know

Skip Pattern:	
<1-8>	Go to CTLNGREASON_A

CTLNGREASON_A

5. Thinking about this last CT exam, was this because of a problem with your health?
   1) Yes
   2) No
   3) Refused
   4) Don't Know

Skip Pattern:	
<1>	End of lung cancer screening survey
<2-4>	Go to CTLNGREASON_B

CTLNGREASON_B

6. Was this as a follow-up test of an earlier test or screening exam?
   1) Yes
   2) No
   3) Refused
   4) Don't Know

Skip Pattern:

<1>	End of lung cancer screening survey
<2-4>	Go to CTLNGREASON_C

CTLNGREASON_C

7. Was this last CT exam part of a routine exam or a screening examination?
    1) Yes
    2) No
    3) Refused
    4) Don't Know
