# Supplementary material for: Protocol to measure validity and reliability of colorectal, breast, cervical and lung cancer screening questions from the 2021 National Health Interview Survey: Methodology and design
Source: PLoS One. 2024 Mar 4;19(3):e0297773. doi: 10.1371/journal.pone.0297773 (PMC10911603; doi:10.1371/journal.pone.0297773)
Supplement: S2 Appendix — (PDF) [file pone.0297773.s002.pdf]

Appendix 2: Decisions related to specific cancer screening exams

Lung Cancer Screening

We will be using Low-Dose CT exams to validate whether a participant received a lung cancer screening exam. Through discussions with local experts, we learned that nearly all LDCT outpatient exams are used for screening purposes. We decided to remove any hospital LDCT exams as those are typically used for diagnostic purposes.

Cervical Cancer Screening

Most recent exam can be validated with combinations of codes and age recommendations. CPT, ICD-9 and HMC codes can be used to verify some of the cervical cancer screening questions. The result of the test would need to be pulled to determine if the exam is screening or diagnostic. If we do not have access to the result of a specific exam, then we will have limitations to determining the reason(s) for the exam. It is also difficult to flag ineligible participants, as some patients who receive hysterectomies continue to receive cervical cancer screenings. LOINC codes were also not utilized to determine whether a participant had received a pap test; LOINC codes are not always available at UW, not accessible at KP, MHS, or HFH.

Colorectal Cancer Screening

We considered all screening colonoscopies with polyp removal as screening exams. We also considered all outpatient FIT/FOBT exams as screening exams; we excluded hospitalized FIT/FOBT exams as these exams are typically diagnostic.

Breast Cancer Screening

We will use EMR codes to validate mammogram exams since MHS does not have access to a radiology information system and the UW Radiology Information System (RIS) includes mostly non-UW patient data. KP has access to a breast cancer RIS and will be using the RIS to determine indication for mammogram exams.
