# Supplementary material for: Protocol to measure validity and reliability of colorectal, breast, cervical and lung cancer screening questions from the 2021 National Health Interview Survey: Methodology and design
Source: PLoS One. 2024 Mar 4;19(3):e0297773. doi: 10.1371/journal.pone.0297773 (PMC10911603; doi:10.1371/journal.pone.0297773)
Supplement: S3 Appendix — (PDF) [file pone.0297773.s003.pdf]

Appendix 3: Questions that cannot be validated

Cost of exam
Since we will not be accessing information on the cost of the tests, this question falls outside of the scope of this study.

Age at time of first screening exam
Since we will only be validating exams that occurred in the past five years, we cannot ensure that their entire screening history for an exam will be included.

Whether their healthcare provider explained exams participant received
We cannot validate as this information is not logged into medical records.

Whether a participant ever had an exam
We cannot validate whether a participant ever had an exam since some exams may fall outside of the five-year time frame. We will look for evidence of correspondence within records if participants respond that they have received an exam and this exam occurred within the past five years. However, if a participant says that they have, and we cannot find records in the past five years, their response would not be invalidated as the exam could have taken place over five years ago or at a separate health care system. If a participant says they have never been screened and our records indicate they have, then we could potentially invalidate their response.

Most important reason why a participant has not received a screening exam
We cannot validate this question since there is no automated data available on why the participant has not received an exam.

Questions we will validate:

How long it has been since their most recent screening exam
We can validate for exams that occurred in the past five years.

We will consider validating a positive answer if the respondent answers affirmative to the screening exam and the electronic medical record has an entry for that exam within X months of the self-report information OR the respondent says there has been no exam within the previous Y months and there is no record of such an exam. All other combinations of responses will count as an invalid response.

Main reason for most recent exam
We will validate if indication for the exam is available in claims codes.

Receipt of cancer screening exams
We can validate for exams that occurred in the past five years.

Whether screening exams required follow-up in the past five years
We could validate if the exam occurred in the past five years.
