# Supplementary material for: Protocol to measure validity and reliability of colorectal, breast, cervical and lung cancer screening questions from the 2021 National Health Interview Survey: Methodology and design
Source: PLoS One. 2024 Mar 4;19(3):e0297773. doi: 10.1371/journal.pone.0297773 (PMC10911603; doi:10.1371/journal.pone.0297773)
Supplement: Attachment — Submitted filename: PLOS ONE response to reviewers letter-20 Dec 2023.docx [file pone.0297773.s004.docx]

20 December 2023

Deepavali A Dhond, Ph.D.
Editorial Office
PLOS ONE

## Dear Dr. Dhond:

This letter serves as a response to the review we received on our original paper, PONE-D-23-39604. I have reproduced the entire decision letter for convenience and to aid in the reviewer(s) to understand the revisions:

PONE-D-23-39604
Protocol to Measure Validity and Reliability of Colorectal, Breast, Cervical and Lung Cancer Screening Questions from the 2021 National Health Interview Survey: Methodology and Design
PLOS ONE

Dear Dr. Kessler,

Thank you for submitting your manuscript to PLOS ONE. Unfortunately, after careful consideration, we have decided that your manuscript does not meet our criteria for publication and must therefore be rejected.

Study Protocols submitted to PLOS ONE should report the study proposal prior to completing participant recruitment or data collection ([https://journals.plos.org/plosone/s/submission-guidelines#loc-study-protocols](https://urldefense.com/v3/__https:/journals.plos.org/plosone/s/submission-guidelines*loc-study-protocols__;Iw!!K-Hz7m0Vt54!lTEU2aSql4gQlY8qZwVD0jpj8W3N6Se266J_n0141nWZtKu1-lkNCCg8QDr0_UDFzD5J6ciXjQEctOw$) ). In this case, we note that patient recruitment has already been completed. Preliminary or pilot data may be included, but only if necessary to support the feasibility of the study or as a proof of principle. As such, this submission does not meet our criteria for Study Protocols.

I am sorry that we cannot be more positive on this occasion, but hope you understand the reason for this decision.

Sincerely,
Deepavali A Dhond, Ph.D.
Editorial Office
PLOS ONE

On behalf of our entire study team, we re-submit this article for your consideration: Protocol to Measure Validity and Reliability of Colorectal, Breast, Cervical and Lung Cancer Screening Questions from the 2021 National Health Interview Survey: Methodology and Design.

**Specifically, the reason given below for the rejection is that protocol papers must be submitted before the completion of participant recruitment and data collection. Our study continues to accrue participants and collect data and will do so for months.**

We have revised our original submission to make clear this effort is ongoing.

We look forward to hearing from the journal and its reviewers.

Respectfully submitted,

Larry Kessler, Sc.D.

Professor

Department of Health Systems and Population Health (HSPOP)

School of Public Health

University of Washington
