# Supplementary material for: Protocol to measure validity and reliability of colorectal, breast, cervical and lung cancer screening questions from the 2021 National Health Interview Survey: Methodology and design
Source: PLoS One. 2024 Mar 4;19(3):e0297773. doi: 10.1371/journal.pone.0297773 (PMC10911603; doi:10.1371/journal.pone.0297773)
Supplement: Peer review history

# Author comment: Author response to previous submission

**DOI:** 10.1371/journal.pone.0297773.r001

21 Dec 2023

Attachment Submitted filename: PLOS ONE response to reviewers letter-20 Dec 2023.docx

# Editor report: Decision Letter 0

**DOI:** 10.1371/journal.pone.0297773.r002

**Authors:** Eugenio Paci

12 Jan 2024

Protocol to Measure Validity and Reliability of Colorectal, Breast, Cervical and Lung Cancer Screening Questions from the 2021 National Health Interview Survey: Methodology and Design

PONE-D-23-42950

Dear Dr. Kessler,

We’re pleased to inform you that your manuscript has been judged scientifically suitable for publication and will be formally accepted for publication once it meets all outstanding technical requirements.

Within one week, you’ll receive an e-mail detailing the required amendments. When these have been addressed, you’ll receive a formal acceptance letter and your manuscript will be scheduled for publication.

An invoice for payment will follow shortly after the formal acceptance. To ensure an efficient process, please log into Editorial Manager at http://www.editorialmanager.com/pone/, click the 'Update My Information' link at the top of the page, and double check that your user information is up-to-date. If you have any billing related questions, please contact our Author Billing department directly at authorbilling@plos.org.

If your institution or institutions have a press office, please notify them about your upcoming paper to help maximize its impact. If they’ll be preparing press materials, please inform our press team as soon as possible -- no later than 48 hours after receiving the formal acceptance. Your manuscript will remain under strict press embargo until 2 pm Eastern Time on the date of publication. For more information, please contact onepress@plos.org.

Kind regards,

Eugenio Paci, MD

Academic Editor

PLOS ONE

Journal requirements:

When submitting your revision, we need you to address these additional requirements.

1. Please ensure that your manuscript meets PLOS ONE's style requirements, including those for file naming. The PLOS ONE style templates can be found at 

https://journals.plos.org/plosone/s/file?id=wjVg/PLOSOne_formatting_sample_main_body.pdf and 

https://journals.plos.org/plosone/s/file?id=ba62/PLOSOne_formatting_sample_title_authors_affiliations.pdf

2. In the ethics statement in the Methods, you have specified that verbal consent was obtained. Please provide additional details regarding how this consent was documented and witnessed, and state whether this was approved by the IRB

3. Please note that funding information should not appear in any section or other areas of your manuscript. We will only publish funding information present in the Funding Statement section of the online submission form. Please remove any funding-related text from the manuscript.

4. Please include your full ethics statement in the ‘Methods’ section of your manuscript file. In your statement, please include the full name of the IRB or ethics committee who approved or waived your study, as well as whether or not you obtained informed written or verbal consent. If consent was waived for your study, please include this information in your statement as well. 

Additional Editor Comments (optional):

The protocol presented in this paper is of relevant interest and I hope the authors will be able to perform the whole work they planned to do. The use of reported data about screening interventions is widespread in many countries but the quality assessment of the reporting rare. The design of a randomized trial to validate the best practices to collect responses and validate them is new and important methodologically also for future developments.

This is certainly a first step. As the authors acknowledge, there are problems not fully addressed in the protocol (for example health literacy or other languages). However this study might be a reference for future investigations.

Finally, as European where screening is offered in organized screening programs I am very interested in the question about previous test and timing of screening. Certainly, a cooperative international research might be a useful initiative (may be already ongoing).

Reviewers' comments:

# Editor report: Acceptance letter

**DOI:** 10.1371/journal.pone.0297773.r003

**Authors:** Eugenio Paci

21 Feb 2024

PONE-D-23-42950 

PLOS ONE

Dear Dr. Kessler, 

I'm pleased to inform you that your manuscript has been deemed suitable for publication in PLOS ONE. Congratulations! Your manuscript is now being handed over to our production team.

At this stage, our production department will prepare your paper for publication. This includes ensuring the following:

* All references, tables, and figures are properly cited

* All relevant supporting information is included in the manuscript submission,

* There are no issues that prevent the paper from being properly typeset

If revisions are needed, the production department will contact you directly to resolve them. If no revisions are needed, you will receive an email when the publication date has been set. At this time, we do not offer pre-publication proofs to authors during production of the accepted work. Please keep in mind that we are working through a large volume of accepted articles, so please give us a few weeks to review your paper and let you know the next and final steps. 

Lastly, if your institution or institutions have a press office, please let them know about your upcoming paper now to help maximize its impact. If they'll be preparing press materials, please inform our press team within the next 48 hours. Your manuscript will remain under strict press embargo until 2 pm Eastern Time on the date of publication. For more information, please contact onepress@plos.org.

If we can help with anything else, please email us at customercare@plos.org.

Thank you for submitting your work to PLOS ONE and supporting open access. 

Kind regards, 

PLOS ONE Editorial Office Staff

on behalf of

Dr. Eugenio Paci 

Academic Editor

PLOS ONE
